# Iron Deficiency in Heart Failure: Cellular Mechanisms and Therapeutic Implications

**DOI:** 10.3390/jcdd12110415

**Published:** 2025-10-22

**Authors:** Anastasios Tsarouchas, Vassilios P. Vassilikos, Dimitrios Mouselimis, Christodoulos E. Papadopoulos, Dimitrios Tachmatzidis, Aikaterini Vassilikou, Constantinos Bakogiannis

**Affiliations:** Third Department of Cardiology, Aristotle University of Thessaloniki, “Hippokration” General Hospital, 54642 Thessaloniki, Greece; tasos.tsarouchas@gmail.com (A.T.);

**Keywords:** heart failure, iron deficiency, intravenous ferrum treatment, anemia

## Abstract

Iron deficiency (ID) is a prevalent comorbidity in heart failure (HF), affecting 37–75% of patients and contributing significantly to symptom burden and adverse outcomes independent of anemia status. Current diagnostic criteria for ID in HF include absolute deficiency (ferritin <100 μg/L) and functional deficiency (ferritin 100–299 μg/L with transferrin saturation <20%). Major clinical trials including AFFIRM-AHF, IRONMAN, HEART-FID, and FAIR-HF2 have demonstrated that intravenous iron therapy, particularly ferric carboxymaltose, reduces HF hospitalizations and improves quality of life and exercise capacity. The 2023 European Society of Cardiology guidelines recommend intravenous ferric carboxymaltose for symptomatic iron-deficient patients with heart failure with reduced ejection fraction. Despite these advances, significant knowledge gaps remain regarding optimal diagnostic approaches, the relationship between ID and ferroptosis in cardiac tissue, and the efficacy of newer iron formulations. This review synthesizes current understanding of ID in HF and highlights emerging therapeutic strategies.

## 1. Introduction

Heart failure (HF) represents a global health challenge, affecting an estimated 64.3 million people worldwide [1]. With an aging population and improved survival from acute cardiovascular events, the prevalence of HF is projected to increase by 46% by 2030, resulting in more than 8 million cases in the United States alone [2,3]. Despite advances in pharmacological and device therapies, HF continues to be associated with significant morbidity, mortality, and healthcare expenditure.

Iron deficiency (ID) has emerged as a key comorbidity in HF, with a prevalence ranging from 37% to 75% depending on the population studied and the definition used [4]. Importantly, ID in HF is associated with reduced exercise capacity, poorer quality of life, increased hospitalization rates, and higher mortality, independent of anemia status [5,6,7,8]. The recognition of ID as a therapeutic target in HF has led to significant research into both the pathophysiological mechanisms and potential treatment strategies.

This review examines the cellular mechanisms underlying iron’s critical role in cardiomyocyte function, the diagnostic criteria for ID in HF, the evidence base for iron supplementation therapy, and emerging therapeutic approaches. By synthesizing current knowledge and highlighting areas for future research, this review aims to provide a comprehensive understanding of ID in HF and its implications for clinical practice.

## 2. Anemia in Heart Failure: It Is Not Just Iron Deficiency

Anemia represents a common comorbidity in HF, with prevalence rates ranging from 15% to 70% depending on the population studied, HF severity, and diagnostic criteria employed [9]. Recent epidemiological studies indicate that approximately 30–50% of patients with chronic HF and up to 80% of those with acute decompensated HF present with anemia [10,11]. The presence of anemia in HF patients is independently associated with increased mortality, hospitalization rates, and diminished quality of life, highlighting the importance of accurate diagnosis and appropriate management.

The development of anemia in HF reflects a complex interplay of pathophysiological mechanisms rather than a single etiological factor. ID represents the predominant cause, accounting for approximately 50–73% of cases [12]. Contemporary research distinguishes between absolute ID (depleted iron stores due to reduced dietary intake, impaired gastrointestinal absorption, or chronic blood loss) and functional ID (adequate iron stores but unavailable for erythropoiesis due to inflammatory processes) [13].

The inflammatory state characteristic of HF promotes hepcidin production, primarily by hepatocytes but also by cardiomyocytes themselves as has been demonstrated [14]. Hepcidin binds to ferroportin, triggering its internalization and degradation, effectively trapping iron within enterocytes, hepatocytes, and macrophages, limiting its availability for erythropoiesis despite adequate total body iron stores. Gastrointestinal edema secondary to right-sided HF further contributes to ID by impairing intestinal absorption, with recent studies demonstrating that up to 57% of HF patients with ID exhibit gastrointestinal mucosal abnormalities [15].

Cardiorenal syndrome represents another significant contributor to anemia in HF. Reduced renal perfusion secondary to decreased cardiac output leads to impaired erythropoietin production [16]. The relationship is further complicated by erythropoietin resistance, as inflammatory cytokines interfere with erythropoietin signaling at the level of erythroid progenitor cells, reducing their responsiveness despite adequate or even elevated erythropoietin levels [16].

Plasma volume expansion often results in pseudoanemia due to hemodilution rather than true red cell deficiency, particularly in patients with acute decompensated HF. Recent studies employing direct measurement of plasma volume have demonstrated that up to 46% of HF patients with apparent anemia actually have pseudoanemia [17].

HF medications, such as renin–angiotensin system blockers or mineralocorticoid receptor antagonists, may further suppress erythropoiesis in some patients [18]. Deficiencies in vitamin B12 and folate, though less common, can also play a role [19], often related to malnutrition in advanced disease stages.

Taken together, anemia in HF is multifactorial and not solely a manifestation of ID. Recognizing its diverse causes is essential to guide appropriate diagnostic workup and avoid ineffective therapy. Importantly, its main cause, iron dysregulation, leads to more than insufficient hematopoiesis; it exerts direct effects on cardiomyocyte biology, as discussed below.

## 3. Cellular Role of Iron in Cardiomyocytes

Mitochondrial function and energy production in cardiomyocytes depend critically on iron availability. Cardiac cells utilize fatty acids as their primary metabolic substrate, generating ATP through a sophisticated sequence of biochemical reactions spanning β-oxidation, the tricarboxylic acid cycle, and oxidative phosphorylation (OXPHOS). Iron serves as an indispensable cofactor for numerous enzymes within these pathways, particularly as a constituent of heme-containing proteins and iron–sulfur (Fe-S) clusters that facilitate electron transport during OXPHOS. Contemporary investigations confirm that disruptions in iron homeostasis profoundly compromise these energy-generating processes and overall mitochondrial performance [6,7].

Experimental models have provided crucial insights into cardiac iron metabolism. Targeted deletion of transferrin receptor 1 (Tfr1), the primary mediator of transferrin-bound iron uptake via receptor-mediated endocytosis, in murine cardiac tissue produces profound metabolic consequences [20]. Xu and colleagues demonstrated that cardiomyocyte-specific Tfr1 knockout resulted in severe mitochondrial dysfunction characterized by impaired fatty acid metabolism, disrupted mitophagy, diminished activity of iron-dependent OXPHOS enzymes, and compensatory upregulation of glycolytic pathways. This metabolic reprogramming proved insufficient to maintain energy homeostasis, culminating in early-onset lethal dilated cardiomyopathy. Notably, aggressive iron supplementation enabled non-Tfr1-mediated iron acquisition and reversed the pathological phenotype in most animals, underscoring iron’s fundamental importance in cardiac function [20].

Beyond the canonical transferrin-mediated pathway, cardiomyocytes possess multiple mechanisms for acquiring non-transferrin bound iron [21,22]. Recent investigations have identified several transmembrane transporters facilitating this process, including divalent metal transporter 1 (DMT1), L-type and T-type calcium channels, and various other metal transport proteins [22]. The regulatory framework governing cardiac iron flux has become increasingly well-characterized, though certain aspects remain incompletely understood. While intestinal epithelial cells and macrophages export iron via ferroportin under hepcidin regulation [23], this iron-regulatory axis operates with tissue-specific modifications in cardiac cells [24].

In vivo studies utilizing genetic knockout approaches have established that cardiomyocytes employ ferroportin for cellular iron efflux, preventing intracellular iron accumulation. Remarkably, cardiac tissue autonomously produces hepcidin, which functions in an autocrine manner to modulate ferroportin activity. This mechanism allows cardiomyocytes to restrict iron export during periods of limited availability, thereby preserving intracellular iron content [14,25]. Recent work has further elucidated this cardiac-specific hepcidin–ferroportin regulatory circuit, demonstrating its responsiveness not only to systemic iron status but also to local tissue oxygenation and inflammatory signaling, providing cardiomyocytes with autonomous control over their iron content independent of systemic iron homeostasis [26].

Haddad and colleagues highlighted the critical role of Iron Regulatory Proteins (IRPs) in orchestrating the hepcidin–ferroportin system according to intracellular iron conditions. IRP1 and IRP2 interact with iron-responsive elements (IREs) located in untranslated regions of specific mRNAs, enhancing Tfr1 mRNA stability while inhibiting ferroportin and ferritin mRNA translation. This coordinated response increases intracellular iron concentration [27]. The system incorporates negative feedback mechanisms, as iron-replete conditions trigger post-translational inactivation of IRP1 and accelerated degradation of IRP2. IRP-deficient mice exhibit normal baseline phenotypes but demonstrate markedly reduced cardiac functional reserve and increased mortality following myocardial infarction. Intravenous iron therapy appeared to counteract these deleterious effects [27].

**Figure 1:**
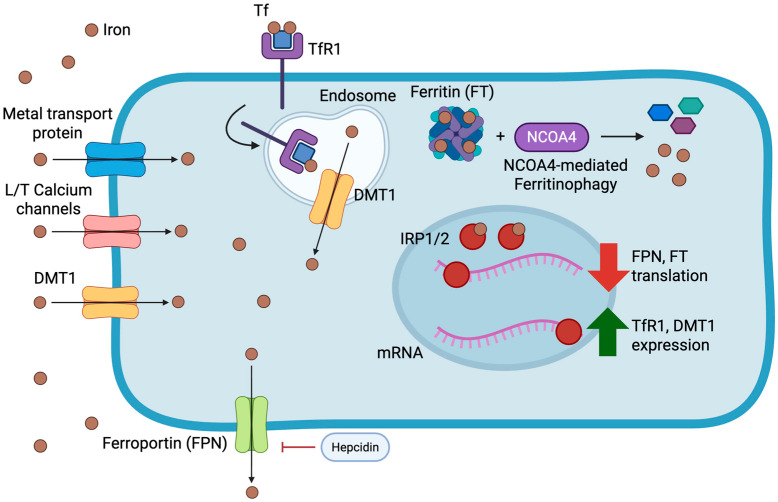
Overview of cardiomyocyte iron metabolism and its regulatory mechanisms. Iron enters cardiomyocytes primarily via transferrin receptor 1 (TfR1)-mediated endocytosis of transferrin-bound iron and through non-transferrin-bound pathways including divalent metal transporter 1 (DMT1), L- and T-type calcium channels, and other metal transport proteins. Intracellular iron is stored within ferritin (FT) and released through NCOA4-mediated ferritinophagy to maintain cytosolic iron availability. Ferroportin (FPN) facilitates iron efflux, and its activity is negatively regulated by hepcidin, which in the heart is also produced locally in an autocrine fashion. Cytosolic iron levels are further balanced by the Iron Regulatory Proteins (IRP1/2), which bind to iron-responsive elements (IREs) on target mRNAs: under iron-depleted conditions, IRP binding stabilizes TfR1 and DMT1 transcripts while repressing ferritin and ferroportin translation; conversely, iron-replete states inactivate IRPs, promoting ferritin synthesis and iron export. Together, these systems preserve myocardial iron homeostasis and prevent both deficiency and overload.

Contemporary advances in iron metabolism research have revealed nuclear receptor coactivator 4 (NCOA4) as a key mediator of ferritinophagy—the selective autophagic degradation of ferritin to mobilize stored iron [28]. This process assumes particular importance during periods of iron limitation and exhibits dysregulation in HF, potentially contributing to the frequently observed discordance between systemic and myocardial iron parameters [29]. All of the aforementioned pathways are illustrated in Figure 1.

Experimental investigations have also revealed the relationship between ID and ferroptosis, an iron-dependent form of regulated cell death characterized by the accumulation of lipid peroxides [30]. In cardiac tissue, ferroptosis has been implicated in ischemia–reperfusion injury and the progression of cardiomyopathy [31]. The complex interplay between ID, iron overload, and ferroptosis represents an emerging area of research with potential therapeutic implications for HF patients [32].

## 4. Iron Deficiency in Heart Failure and the Role of Intravenous Ferrum Treatment

As stated above, ID is a significant comorbidity in HF, whose deleterious effects on patient quality of life, exercise capacity, and risk of hospitalizations have been repeatedly demonstrated [5,6,7,8]. Several pathophysiological mechanisms have been proposed to account for the high prevalence of ID in this population, including impaired gastrointestinal absorption due to gut edema or concomitant proton pump inhibitor therapy, increased blood loss in the context of antiplatelet or anticoagulant use, systemic inflammation, and reduced dietary intake [33,34]. However, none of these mechanisms alone sufficiently explains the magnitude of ID observed in HF, and the etiology remains incompletely understood.

### 4.1. Defining and Detecting Iron Deficiency in Heart Failure

Although the origins of ID in HF remain incompletely explained, progress has been made in standardizing its clinical definition and detection. The diagnostic criteria of ID in HF have evolved significantly over time, with recognition that traditional laboratory parameters used in the general population may have limited sensitivity and specificity in the HF setting due to the confounding effects of inflammation. Current guidelines from the European Society of Cardiology (ESC) define ID in HF as serum ferritin <100 μg/L (absolute ID) or serum ferritin 100–299 μg/L with transferrin saturation (TSAT) <20% (functional ID) [35].

However, recent research suggests these criteria may require refinement. A landmark study comparing various laboratory parameters against the gold standard of bone marrow iron staining found that serum ferritin <100 μg/L alone had a sensitivity of only 82% and specificity of 72% for identifying ID [9]. Alternative biomarkers including soluble transferrin receptor concentration and hepcidin levels have emerged as promising diagnostic tools that may be less affected by inflammation than traditional parameters [11].

Even with the current criteria for defining ID, treating it in HF patients has been shown to improve outcomes. As will be described in detail below, multiple randomized controlled trials have demonstrated improvements in functional capacity, quality of life, and reduction in HF hospitalizations with intravenous iron therapy in iron-deficient HF patients [36,37].

### 4.2. Clinical Trial Evidence

The recognition of ID as a therapeutic target in HF has led to several randomized controlled trials evaluating the efficacy and safety of intravenous (IV) iron supplementation (Table 1). Oral iron has shown limited efficacy in HF patients, likely due to poor absorption related to hepcidin upregulation and gastrointestinal edema [38].

The FAIR-HF trial, published in 2009, was the first major study to demonstrate benefits of IV iron therapy in HF. This trial randomized 459 patients with HF with reduced ejection fraction (HFrEF) and ID to receive either IV ferric carboxymaltose or placebo. At 24 weeks, patients in the treatment group showed significant improvements in self-reported Patient Global Assessment, New York Heart Association (NYHA) functional class, 6 min walk test distance, and quality of life [36].

The CONFIRM-HF trial subsequently demonstrated sustained benefits of IV ferric carboxymaltose over a longer 52-week period, with significant improvements in 6 min walk test distance (primary endpoint) and reductions in the risk of hospitalization for worsening HF [37].

Later on, the AFFIRM-AHF trial demonstrated that treatment with IV ferric carboxymaltose in iron-deficient patients hospitalized for acute HF reduced the risk of HF hospitalizations (rate ratio 0.74, 95% CI 0.58–0.94, *p* = 0.013), although it did not significantly reduce the primary composite endpoint of total HF hospitalizations and cardiovascular death [39].

The IRONMAN trial, published in 2023, evaluated IV ferric derisomaltose in patients with HFrEF and ID, showing a modest but non-significant reduction in the primary endpoint of recurrent HF hospitalizations and cardiovascular death (rate ratio 0.82, 95% CI 0.66–1.02, *p* = 0.070) [40].

The HEART-FID trial, the largest study of IV iron in HF to date, investigated ferric carboxymaltose in 3065 patients with HFrEF and ID. Results published in 2023 showed no significant difference in the primary endpoint of time to first HF hospitalization or cardiovascular death (hazard ratio 0.95, 95% CI 0.84–1.07, *p* = 0.40), although improvements in 6 min walk distance were observed [41].

Most recently, the FAIR-HF2 trial evaluated intravenous ferric carboxymaltose (FCM) in 1105 ambulatory patients with symptomatic HF, left ventricular ejection fraction (LVEF) ≤ 45%, and ID [42]. Published in 2025, this large multicenter, double-blind, randomized study tested three co-primary endpoints, namely the time to cardiovascular death or first HF hospitalization, total HF hospitalizations, and the time to cardiovascular death or first HF hospitalization in patients with transferrin saturation <20%.

Over a median follow-up of 16.6 months, FCM was associated with fewer events of cardiovascular death or first HF hospitalization compared with placebo (HR 0.79, 95% CI 0.63–0.99; *p* = 0.04). However, owing to multiplicity adjustment using the Hochberg–Benjamini procedure, this did not reach formal statistical significance, rendering the trial neutral for its primary efficacy endpoint.

Total HF hospitalizations were likewise lower but not statistically significant (rate ratio 0.80, 95% CI 0.60–1.06; *p* = 0.12), and results were consistent in the subgroup with transferrin saturation <20% (HR 0.79, 95% CI 0.61–1.02; *p* = 0.07). Among the secondary outcomes, there were modest improvements in 6 min walk distance (+10.7 m; 95% CI −1.4 to 22.9) and EQ-5D quality-of-life score (+0.03; 95% CI 0.01 to 0.06), as well as in patient-reported global well-being (odds ratio 0.25; 95% CI 0.17–0.37;). Change in NYHA functional class was not significant (odds ratio 0.69; 95% CI 0.37–1.29). Serious adverse events occurred at similar rates between groups (48.2% vs. 49.9%; *p* = 0.61).

Overall, FAIR-HF2 demonstrated a detectable but statistically non-significant reduction in HF events, confirming the safety of long-term FCM therapy and providing effect estimates consistent with prior outcome trials such as AFFIRM-AHF and IRONMAN. A pre-specified sex-specific analysis, discussed below, revealed potential differences in treatment response between men and women.

### 4.3. Meta-Analyses and Pooled Analyses

Several comprehensive meta-analyses have evaluated the collective evidence from these trials. A 2024 meta-analysis of 14 randomized controlled trials including 6614 patients found that IV iron therapy reduced HF hospitalizations (risk ratio 0.74, 95% CI 0.61–0.89) and improved quality of life and exercise capacity, with no significant effect on all-cause or cardiovascular mortality [43].

A more recent meta-analysis published in 2025 included the HEART-FID and FAIR-HF2 trials, reaching a total of over 7000 patients randomized to intravenous iron therapy or placebo. The results showed in a significant reduction in the combined endpoint of cardiovascular mortality and HF hospitalizations at 12 months (risk ratio 0.72, 95% CI 0.55–0.89), mainly driven by the reduction in HF hospitalizations (risk ratio 0.69, 95% CI 0.48–0.88) with a non-significant trend towards reduction in cardiovascular mortality evaluated separately (risk ratio 0.80, 95% CI 0.61–1.03) [44].

Pooled analyses have also provided insights into specific patient subgroups. A 2023 analysis of individual patient data from four major trials found that the benefits of IV iron were consistent across subgroups defined by age, HF etiology, and baseline hemoglobin levels [45]. Somewhat expectedly, patients with more severe and above all functional ID (TSAT <15%) appeared to derive the greatest benefit among trial participants.

### 4.4. Guideline Recommendations

The evolving evidence base has informed guideline recommendations. The 2016 ESC guidelines for HF gave a Class IIa recommendation (Level of Evidence A) for IV ferric carboxymaltose in symptomatic patients with HFrEF and ID to improve symptoms and reduce HF hospitalizations [46].

The 2021 ESC guidelines maintained this recommendation for HFrEF and expanded it to include a Class IIb recommendation (Level of Evidence B) for patients with HF with mildly reduced ejection fraction (HFmrEF, LVEF 41–49%) [35]. The guidelines define ID as serum ferritin <100 μg/L or ferritin 100–299 μg/L with TSAT < 20%.

The 2023 focused update of the ESC guidelines strengthened the recommendation for HFrEF to Class I (Level of Evidence A), specifically recommending IV ferric carboxymaltose for symptomatic patients (NYHA class II–IV) with HFrEF and ID to reduce the risk of HF hospitalization [47].

The American College of Cardiology/American Heart Association guidelines have been more conservative, not yet including specific recommendations for iron therapy, though their most recent scientific statement acknowledges the potential benefits in selected patients [48].

### 4.5. Dosing Strategies and Formulations

Several intravenous iron formulations have been evaluated in HF, with ferric carboxymaltose being the most extensively studied. This formulation was the first to allows for rapid administration of sizeable doses (up to 1000 mg in a single infusion), reducing the number of medical visits required to achieve adequate iron substitution [49]. Ferric derisomaltose (previously known as iron isomaltoside) has similar pharmacokinetic properties and has demonstrated comparable efficacy in the IRONMAN trial [40].

Dosing strategies have evolved from fixed regimens to more individualized approaches based on body weight and hemoglobin levels. The Ganzoni formula, which calculates iron deficit based on actual and target hemoglobin, body weight, and iron stores, has been largely replaced by simplified dosing tables in clinical practice [50]. Recent evidence suggests that higher cumulative doses may be associated with greater clinical benefits, though the optimal maintenance strategy remains unclear [51].

The timing of iron administration has also been investigated, with the AFFIRM-AHF trial demonstrating benefits when initiated during hospitalization for acute HF [39]. This approach may capitalize on the heightened inflammation and iron sequestration characteristic of acute decompensation, though further research is needed to optimize timing strategies.

### 4.6. Special Populations

The efficacy of intravenous iron therapy appears consistent across various HF phenotypes, though with varying levels of evidence. The following subsections review available data on ID and intravenous iron treatment in specific patient groups, including those with heart failure with preserved ejection fraction (HFpEF), chronic kidney disease (CKD), left ventricular assist devices (LVAD), and potential sex-related differences.

#### 4.6.1. Heart Failure with Preserved Ejection Fraction

Evidence from a recent meta-analysis including 1877 patients with HFpEF indicates that ID is at least as common in HFpEF as in overall HF populations, with a pooled prevalence of approximately 59% (95% CI 52–65%) [52]. ID in HFpEF contributes substantially to reduced exercise capacity, poorer quality of life, and overall morbidity, mirroring the detrimental impact previously established in HFrEF [53]. However, a clear evidence gap emerges when evaluating the therapeutic benefit of intravenous iron in this subgroup.

The FAIR-HFpEF trial was a multicentre, randomized, double-blind study designed to compare intravenous ferric carboxymaltose (FCM) with placebo in 200 patients with symptomatic HFpEF and ID [54]. The trial was terminated early after enrolling only 39 patients (median age 80 years, 62% women) due to slow recruitment. Despite its limited size, FAIR-HFpEF demonstrated a significantly greater improvement in 6 min walk distance with FCM compared to placebo (least-square mean difference 49 m, 95% CI 5–93; *p* = 0.029), and fewer serious adverse events (rate ratio 0.27, 95% CI 0.07–0.96; *p* = 0.043) [52]. Changes in NYHA class and quality-of-life metrics were not statistically significant, reflecting the study’s limited power.

Even fewer studies have explored whether the underlying mechanisms of ID differ between HF phenotypes. It can be hypothesized that systemic inflammation and microvascular dysfunction may exert a greater influence in HFpEF [55,56]. This distinction, although largely speculative, underscores the need for mechanistic and therapeutic studies specifically tailored to HFpEF.

#### 4.6.2. Chronic Kidney Disease

Patients with kidney disease represent another important subgroup, as both HF and ID are common in this population. In the IRON-CKD trial, 75 non-anemic patients with CKD and HFrEF were randomized to ferric carboxymaltose or placebo. The study did not demonstrate improvements in peak VO_2_, 6 min walk distance, or muscle strength at 4 or 12 weeks. While IV iron was safe, the findings suggest no clear functional benefit in this small cohort, and larger studies are needed to clarify potential effects in CKD-HF populations [57]. Thus, caution is warranted in patients with advanced kidney disease due to potential iron overload and oxidative stress.

#### 4.6.3. LVAD

Patients with durable left ventricular assist devices (LVADs) frequently develop ID, yet evidence to guide treatment remains scarce. In a retrospective cohort study of 213 LVAD recipients, of whom 70 received intravenous iron sucrose, treatment was associated with significant increases in hemoglobin and improvements in New York Heart Association (NYHA) functional class over approximately 12 weeks, without excess adverse events [58]. While these findings suggest potential benefits of iron repletion in this population, randomized trials are needed to confirm effects on exercise capacity, quality of life, right ventricular function, and device-specific outcomes such as pump thrombosis or infection.

### 4.7. Sex-Related Outcome Differences

Gender-specific analyses have revealed notable and sometimes unexpected patterns regarding both the prevalence and therapeutic response of iron deficiency in HF. While women with HF are more frequently affected by iron deficiency than men [59], data on sex-specific outcomes of intravenous iron therapy have only recently emerged.

In pooled analyses of contemporary outcome trials, sex appeared to modify the effect of intravenous iron supplementation. In a sex-focused analysis of the FAIR-HF2 trial men receiving FCM experienced a significant reduction in cardiovascular death or first hospitalization for HF (HR 0.74, 95% CI 0.57–0.95, *p* = 0.016), whereas women derived no prognostic benefit (HR 1.07, 95% CI 0.63–1.82, *p* = 0.80) [60]. Interestingly, symptomatic improvement was more pronounced among women: those treated with FCM reported greater gains in EQ-5D quality-of-life scores and self-perceived well-being compared with men, paralleled by a larger rise in hemoglobin. Despite these symptomatic benefits, numerically higher all-cause mortality and adverse-event rates were noted in women, suggesting possible sex-specific differences in the biological handling or safety profile of intravenous iron.

Earlier outcome trials had already hinted at a similar pattern. In AFFIRM-AHF, ferric carboxymaltose reduced HF hospitalizations in men (rate ratio 0.64, 95% CI 0.46–0.89) but not in women (RR 1.05, 95% CI 0.72–1.53) [39], and in HEART-FID, the hazard ratio for the key secondary composite endpoint favored men (HR 0.86, 95% CI 0.74–1.00) but not women (HR 1.11, 95% CI 0.86–1.43) [41]. Conversely, IRONMAN reported no meaningful sex interaction [40]. Also mentioned above, the most recent meta-analysis on iron supplementation in HFrEF including 7175 patients identified sex as the only significant interaction subgroup: women, on average, did not experience prognostic improvement (RR 0.98, 95% CI 0.75–1.26) [44].

Potential biological explanations for these differences involve sex-specific regulation of iron metabolism, erythropoiesis, and hormonal status. Estrogen modulates intracellular iron homeostasis by suppressing hepcidin synthesis, maintaining ferroportin activity, and enhancing iron export from enterocytes and macrophages [61]. With the decline in estrogen after menopause, hepatic hepcidin levels rise, limiting iron mobilization and potentially blunting the response to intravenous supplementation. Given that the mean age of women in FAIR-HF2 was well beyond the average menopausal transition, it is conceivable that reduced estrogen availability and consequent hepcidin activation may have altered iron utilization or distribution in this population. Experimental models also suggest that estrogen deficiency increases hepatic hepcidin expression and lowers circulating iron, lending support to this hypothesis [62].

Another consideration relates to possible differences in myocardial iron uptake and storage. Small imaging studies have suggested that intravenously administered FCM can transiently increase labile iron in myocardial tissue, raising the theoretical risk of oxidative stress or toxicity with repeated dosing [63]—an effect that might differ by sex, although this remains unproven.

Overall, the current evidence indicates that while intravenous iron supplementation improves symptoms and quality of life in both sexes, its prognostic benefit may be limited to men. Whether these findings are driven by biological factors, hormonal status, or unmeasured confounders requires confirmation in larger, sex-balanced prospective trials. Dedicated studies in pre- and post-menopausal women are warranted to clarify the influence of hormonal regulation on iron metabolism and treatment response in HF.

### 4.8. Safety Profile

The safety profile of intravenous iron therapy in HF has been reassuring across multiple trials. Hypersensitivity reactions with modern IV iron formulations are rare but not negligible—RCT data indicate serious or severe reactions occur in approximately 0.1–1% of patients [64]. Other adverse events, including injection site reactions and transient phosphate decreases, are generally mild and self-limiting.

Theoretical concerns about iron overload, oxidative stress, and increased infection risk have not been substantiated in clinical trials. A 2023 updated meta-analysis of randomized trials involving nearly 5000 patients with HF and ID found no excess risk of infections or cardiovascular events with intravenous iron therapy [65]. These findings are consistent with earlier systematic reviews in both HF and chronic kidney disease populations, supporting the overall safety of intravenous iron. Among randomized trials evaluating long-term iron therapy in HF, IRONMAN offers the most extended follow-up to date: patients received ferric derisomaltose over a median of 2.7 years, with some individuals followed for up to approximately 5.4 years in real-world practice contexts. Crucially, long-term exposure was not associated with increased adverse events [40]. Ongoing registry studies will provide further insights into real-world safety with prolonged use.

### 4.9. Future Directions

Despite significant advances, several knowledge gaps remain. The optimal frequency and duration of iron therapy, particularly for maintenance dosing, require further investigation. Additionally, the development of reliable, non-invasive methods to assess myocardial iron status represents another critical area for research. Current diagnostic criteria based on serum ferritin and TSAT may not accurately reflect cardiac iron content, potentially leading to suboptimal patient selection [24]. Novel imaging techniques, including T2* cardiac magnetic resonance imaging, show promise but require validation in HF populations.

The potential role of oral iron formulations continues to be explored, despite mixed results from the IRONOUT-HF trial [38]. Newer formulations with enhanced absorption, such as sucrosomial iron, have shown promising results in small studies and may offer a more convenient alternative for selected patients [66,67,68].

## 5. Conclusions

ID is a common and clinically significant comorbidity in HF that contributes to symptom burden and adverse outcomes independent of anemia status. The recognition of ID as a therapeutic target has led to significant advances in understanding its pathophysiology and developing effective treatment strategies.

Intravenous iron therapy, particularly with ferric carboxymaltose, has demonstrated benefits in reducing HF hospitalizations and improving quality of life and exercise capacity in patients with HFrEF and ID. These findings have been reflected in strengthened guideline recommendations.

Future research should focus on refining diagnostic approaches, exploring the relationship between iron metabolism and ferroptosis, evaluating newer iron formulations, and establishing optimal long-term management strategies. By addressing these knowledge gaps, the field can continue to advance toward more personalized and effective approaches to managing ID in HF.

## Figures and Tables

**Table 1:** Summary of major randomized controlled trials investigating intravenous iron therapy in heart failure.

Trial	Year	Sample Size	Iron Formulation	Population	Primary Endpoint	Outcome
FERRIC-HF	2008	35	Iron sucrose	Symptomatic CHF (NYHA II–III), with or without anemia	Peak VO_2_	Improved peak VO_2_, NYHA class, and QoL; benefits independent of hemoglobin change
FAIR-HF	2009	459	Ferric carboxymaltose	Ambulatory HFrEF with iron deficiency	Patient Global Assessment, NYHA class	Enhanced symptoms, QoL, and 6 min walk distance
CONFIRM-HF	2015	304	Ferric carboxymaltose	Ambulatory HFrEF, 52-week follow-up	6MWD at 24 weeks	Increased 6MWD and reduced HF hospitalizations
AFFIRM-AHF	2020	1132	Ferric carboxymaltose	Post-acute HFrEF	Composite: HF hospitalizations + CV death	Fewer HF hospitalizations; no significant difference in CV mortality
IRONMAN	2023	1137	Ferric derisomaltose	Stable ambulatory HFrEF	Composite: recurrent HF hospitalizations + CV death	Numerical reduction in events; did not reach statistical significance
HEART-FID	2023	3065	Ferric carboxymaltose	HFrEF, largest trial to date	Time to first HF hospitalization or CV death	No significant difference observed in primary outcome
FAIR-HF2	2025	1105	Ferric carboxymaltose	Ambulatory HFrEF, LVEF ≤45%	CV death or first HF hospitalization, total HF hospitalizations, CV death or first HF hospitalization in subgroup of patients with TSAT < 20%	Relative reduction in primary outcome (HR 0.79); predefined statistical threshold not achieved; QoL significantly improved
FAIR-HFpEF	2024	39 (terminated early)	Ferric carboxymaltose	HFpEF	6MWD	Increased 6MWD and reduced adverse events
RESAFE-HF	2024	96	Ferric carboxymaltose	Ambulatory HFrEF with iron deficiency, all with CIEDs	Arrhythmic burden (nsVTs, device therapies, Holter markers)	Reduced arrhythmias; improved LV function, QoL, exercise capacity, and iron indices; no FCM-related safety issues

CIED—Cardiac implantable electronic device, CV—Cardiovascular, FCM—Ferric carboxymaltose, HF—Heart failure, HFpEF—Heart failure with preserved ejection fraction, HFrEF—Heart failure with reduced ejection fraction, LVEF—Left ventricular ejection fraction, nsVT—Non-sustained ventricular tachycardia, NYHA—New York Heart Association functional classification, QoL—Quality of life, TSAT—Transferrin saturation, 6MWD—6 min walk distance.

## Data Availability

No new data were created or analyzed in this study.
